# Coordinated inflammatory macrophage and vascular smooth muscle cell remodeling signatures in human atherosclerosis: An integrative single‐cell and bulk transcriptomic analysis

**DOI:** 10.1002/ccs3.70106

**Published:** 2026-09-03

**Authors:** Xinyu Dong, Xuesong Hu, Qi Liu, Yuhao Dong, Yan Xuan, Muning Wang, Siyao Chang, Tianyi Lu, Zhiyang Han

**Affiliations:** ^1^ Department of Vascular Surgery the First Affiliated Hospital of Harbin Medical University Harbin Heilongjiang China; ^2^ Department of Pediatrics The Sixth Affiliated Hospital of Harbin Medical University Harbin Heilongjiang China; ^3^ Department of Medical Laboratory Science and Technology Harbin Medical University Daqing Harbin Heilongjiang China; ^4^ Harbin Medical University Harbin Heilongjiang China; ^5^ Department of Breast and Vascular Surgery Harbin the First Hospital Harbin Heilongjiang China; ^6^ Department of Vascular Surgery Harbin Fifth Hospital Harbin Heilongjiang China; ^7^ School of Public Health Harbin Medical University Harbin Heilongjiang China

**Keywords:** atherosclerosis, ligand‐receptor analysis, macrophages, single‐cell RNA sequencing, transcriptomics, vascular smooth muscle cells

## Abstract

Atherosclerotic plaque progression is shaped by coordinated inflammatory and remodeling programs involving immune cells and vascular wall cells. Inflammatory macrophage activation and vascular smooth muscle cell (VSMC) phenotypic remodeling are central features of human atherosclerosis, but their transcriptomic relationships during plaque progression remain incompletely characterized. This study integrated single‐cell and bulk transcriptomic datasets to examine highly inflammatory macrophage states, VSMC remodeling‐related transcriptional programs, and candidate ligand‐receptor expression patterns in human atherosclerotic plaques. Human atherosclerotic plaque single‐cell RNA sequencing data from GSE260657 and bulk transcriptomic data from GSE28829 were analyzed. After quality control, 7628 cells were retained for single‐cell analysis. Major cell types were annotated using canonical markers, followed by reclustering of macrophages and VSMC‐related cells. Functional module scoring, differential expression analysis, Gene Ontology biological process enrichment, and Kyoto Encyclopedia of Genes and Genomes pathway analyses were performed to characterize macrophage transcriptional states. Slingshot was applied to infer VSMC pseudotime ordering. CellChat and NicheNet were used to prioritize candidate ligand‐receptor expression patterns and ligand‐associated VSMC target gene programs. External bulk transcriptomic analysis was performed to examine whether single‐cell‐derived inflammatory and remodeling signatures were represented at the tissue‐transcriptome level during plaque progression. Macrophage reclustering identified a highly inflammatory macrophage state characterized by prominent inflammatory activation, cytokine‐response, and stress‐response features. Genes upregulated in this population were enriched in pathways related to tumor necrosis factor (TNF) response, nuclear factor kappa B signaling, leukocyte activation, cytokine signaling, lipid and atherosclerosis, toll‐like receptor signaling, and inflammasome‐associated inflammation. VSMC reclustering revealed contractile VSMCs, PTHLH+ synthetic VSMCs, KRT7+ VSMC‐like cells, interferon‐responsive VSMCs, pericyte‐like mural cells, and osteogenic/modulated VSMCs. Pseudotime analysis showed a broad contractile‐to‐osteogenic/modulated transcriptional continuum accompanied by increased expression of remodeling‐associated genes and selected inflammatory or remodeling‐associated receptor genes. CellChat and NicheNet analyses prioritized candidate ligand‐receptor and ligand‐associated target gene expression patterns involving SPP1–CD44, TNF–TNFRSF1A, IL1B–IL1R1/IL1RAP, MIF–ACKR3, PDGFB–PDGFRB, and FN1–SDC1/ITGB1. In GSE28829, inflammatory macrophage‐, osteogenic/modulated VSMC‐, candidate ligand‐receptor expression‐, SPP1–CD44 candidate axis‐, and NicheNet‐prioritized target program‐related signatures were more prominent in advanced plaques and were positively correlated with each other. This integrative transcriptomic analysis identified a highly inflammatory macrophage state and a VSMC remodeling continuum in human atherosclerotic plaques. Candidate ligand‐receptor and ligand‐associated target gene expression patterns linked inflammatory macrophage activation with osteogenic/modulated VSMC remodeling at the computational level. External bulk data further showed coordinated enrichment of inflammatory and remodeling signatures in advanced plaques. These findings provide a descriptive and hypothesis‐generating transcriptomic framework for understanding inflammatory macrophage activation and VSMC remodeling in human atherosclerosis.

## Graphical abstract

Integrative single‐cell and bulk transcriptomic analyses identified a highly inflammatory macrophage state and a vascular smooth muscle cell (VSMC) remodeling continuum in human atherosclerotic plaques. Candidate ligand‐receptor and ligand‐associated target gene expression patterns linked inflammatory macrophage activation with osteogenic/modulated VSMC remodeling at the computational level. External bulk data further showed coordinated enrichment of inflammatory and remodeling signatures in advanced plaques.

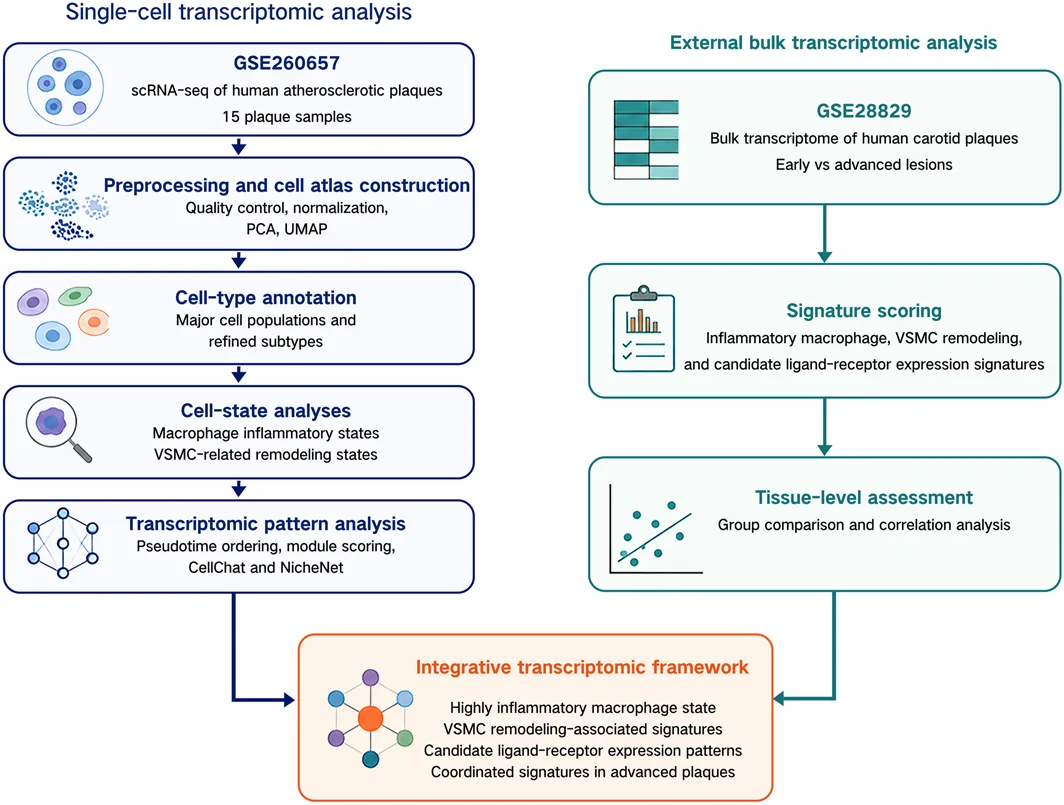

## 1 INTRODUCTION

Atherosclerosis is the major pathological basis of coronary artery disease, ischemic stroke, and peripheral artery disease.^1^ Its development involves lipid deposition, endothelial activation, immune‐cell recruitment, vascular smooth muscle cell (VSMC) phenotypic remodeling, cell death, and extracellular matrix reorganization.^2^, ^3^ Inflammation is now recognized as a central process throughout the life cycle of atherosclerotic plaques from lesion initiation to plaque progression and destabilization.^1^, ^4^ Among immune‐cell populations within plaques, macrophages play critical roles in lipid uptake, foam‐cell formation, inflammatory cytokine production, necrotic‐core expansion, and matrix degradation.^1^, ^4^ Therefore, plaque progression is shaped by coordinated inflammatory activation and structural remodeling within a complex vascular microenvironment.^5^

Single‐cell RNA sequencing (scRNA‐seq) has provided an opportunity to dissect the cellular heterogeneity of human atherosclerotic plaques at high resolution. Recent single‐cell studies have revealed diverse macrophage, endothelial cell, VSMC, and stromal cell states in human lesions as well as disease‐relevant vascular cell states that are difficult to resolve using bulk transcriptomic approaches.^6^, ^7^ In parallel, VSMC plasticity has emerged as a key determinant of plaque biology. During atherogenesis, VSMCs can transition from a contractile phenotype toward synthetic, osteogenic/chondrogenic, fibroblast‐like, macrophage‐like, and inflammatory‐response states.^2^, ^3^, ^7^ These phenotypic transitions are closely associated with fibrous‐cap formation, extracellular matrix remodeling, vascular calcification, and plaque stability.^2^, ^3^, ^5^ Therefore, examining macrophage inflammatory activation together with VSMC remodeling states may help clarify how immune and vascular‐wall programs co‐occur during human plaque progression.

Although inflammatory macrophage activation and VSMC phenotypic modulation have each been extensively studied, their coordinated transcriptional patterns in human atherosclerotic plaques remain incompletely characterized. In particular, it remains important to define how inflammatory macrophage programs are represented in relation to osteogenic/modulated VSMC states, candidate ligand‐receptor expression patterns, and tissue‐level plaque progression. Computational approaches, such as ligand‐receptor inference and ligand‐target prediction, can help prioritize candidate ligand‐receptor and ligand‐associated target gene expression patterns,^8^, ^9^ but these analyses should be interpreted as hypothesis‐generating transcriptomic associations rather than direct evidence of cell‐specific signaling directionality or causal intercellular regulation.

In this study, we integrated human plaque scRNA‐seq data with an external bulk transcriptomic dataset to characterize coordinated inflammatory macrophage and VSMC remodeling signatures in human atherosclerosis. We first constructed a single‐cell atlas of human atherosclerotic plaques and defined major macrophage and VSMC‐related transcriptional states. We then focused on a highly inflammatory macrophage state and osteogenic/modulated VSMC states, examined VSMC pseudotime organization, and inferred candidate ligand‐receptor and ligand‐associated target expression patterns.^8^, ^9^, ^10^ Finally, we assessed whether single‐cell‐derived inflammatory and remodeling signatures were represented in advanced plaques using an external bulk transcriptomic dataset. Through this framework, our study aimed to provide a descriptive and integrative transcriptomic analysis of macrophage inflammatory activation, VSMC remodeling, and coordinated plaque‐associated signatures in human atherosclerosis.

## 2 MATERIALS AND METHODS

### 2.1 Data sources

The human atherosclerotic plaque scRNA‐seq dataset GSE260657 and the bulk transcriptomic dataset GSE28829 were downloaded from the Gene Expression Omnibus (GEO) database.^11^, ^12^, ^13^ GSE260657 contains scRNA‐seq profiles of human carotid atherosclerotic plaques across atherosclerosis progression. In the present study, 15 human plaque samples from this dataset were selected for downstream analysis. GSE28829 contains gene expression profiles from 13 early and 16 advanced human carotid atherosclerotic plaques and was used as an external bulk dataset to examine whether the single‐cell‐derived inflammatory and remodeling signatures were represented at the tissue‐transcriptome level during plaque progression.

### 2.2 Single‐cell RNA sequencing data preprocessing and quality control

scRNA‐seq data were processed using R and the Seurat package.^14^, ^15^ For each sample, the expression matrix was imported and converted into a Seurat object. Quality control was performed based on the number of detected genes per cell, total unique molecular identifier counts, and the percentage of mitochondrial gene expression, which were calculated using the nFeature_RNA, nCount_RNA, and percent.mt metrics, respectively. Cells with 200 < nFeature_RNA < 6000 and percent.mt < 10% were retained for downstream analysis. Cells with extremely low gene counts, excessively high gene counts, or abnormally elevated mitochondrial gene proportions were removed to reduce the potential influence of low‐quality cells and putative doublets. After quality control, 7628 cells and 28,656 genes were retained for downstream analysis.

The filtered expression matrix was normalized using the NormalizeData function. Highly variable genes were identified using FindVariableFeatures, followed by data scaling with ScaleData. Principal component analysis was then performed based on the highly variable genes. According to the elbow plot and the proportion of variance explained by principal components, the first 20 principal components (PCs 1–20) were selected for downstream dimensionality reduction and clustering analyses. Cell clustering was performed using FindNeighbors and FindClusters, with the clustering resolution set to 0.5. Uniform manifold approximation and projection (UMAP) was used for two‐dimensional visualization of the single‐cell transcriptomic landscape. Major cell populations were subsequently annotated based on canonical marker genes.

### 2.3 Cell‐type annotation

Major cell types were annotated according to canonical marker genes and cluster‐specific expression patterns. Macrophages were identified based on the expression of CD68, LYZ, LST1, C1QA, C1QB, C1QC, and APOE. VSMCs were annotated using ACTA2, MYH11, TAGLN, CNN1, MYL9, and MYLK. Endothelial cells were defined by PECAM1, VWF, KDR, CDH5, and CLDN5. T cells/natural killer (NK) cells were identified by CD3D, CD3E, TRAC, NKG7, GNLY, and GZMB. B cells and plasma cells were annotated using MS4A1, CD79A, JCHAIN, and MZB1. Mast cells were identified according to CPA3, TPSAB1, TPSB2, MS4A2, KIT, and HDC. Stromal and mural cell populations were annotated based on COL3A1, DCN, LUM, PDGFRA, PDGFRB, MCAM, CSPG4, and NOTCH3.

### 2.4 Reclustering of macrophages and VSMCs

To further characterize the heterogeneity of plaque macrophages and VSMCs, the annotated macrophage and VSMC populations were extracted separately and reclustered. Macrophage subclusters were annotated according to marker genes, functional module scores, and dominant transcriptional features. Six macrophage subpopulations were identified, including highly inflammatory macrophages, CXCL10+ macrophages, MMP+ plaque‐active macrophages, ACKR3+ intermediate macrophages, CX3CR1+ resident‐like macrophages, and proliferating macrophages.

The highly inflammatory macrophage population was defined as a macrophage state characterized primarily by inflammatory activation, cytokine‐response, and stress‐response transcriptional features, with representative genes including IL1B, NFKBIA, TNFAIP3, CXCL2, CXCL8, NLRP3, PTGS2, and SERPINB2. This annotation reflected the dominant inflammatory and stress‐response characteristics of this cluster and was not intended to define a new macrophage lineage or a structurally distinct macrophage phenotype.

VSMC‐related cells were also extracted and reclustered to define distinct VSMC phenotypic states. Seven initial VSMC‐related populations were identified, including contractile VSMCs, PTHLH+ synthetic VSMCs, KRT7+ VSMC‐like cells, interferon (IFN)‐responsive VSMCs, pericyte‐like mural cells, osteogenic/modulated VSMCs, and endothelial‐like contaminating cells. The endothelial‐like contaminating population was excluded from subsequent VSMC pseudotime analysis. The remaining six VSMC‐related subpopulations were retained for downstream analyses of VSMC phenotypic remodeling.

### 2.5 Functional module scoring and differential expression analysis

Functional module scores were calculated at the single‐cell level using the AddModuleScore function in Seurat. For macrophages, module scores related to inflammatory activation, nuclear factor kappa B (NF‐κB)/tumor necrosis factor (TNF)/IL1 signaling, inflammasome/nucleotide‐binding oligomerization domain (NOD)‐like receptor signaling, lysosome/autophagy, and lipid‐atherosclerosis programs were calculated. These scores were used to summarize the dominant inflammatory, stress‐response, metabolic, and plaque‐associated transcriptional features of macrophage subpopulations.

For VSMCs, module scores related to the contractile phenotype, osteogenic/modulated phenotype, and refined macrophage‐responsive receptor program were calculated. To reduce circularity in pseudotime‐based receptor analysis, intrinsic VSMC phenotypic switching markers, including CD44, FN1, VCAN, and podoplanin (PDPN), were excluded from the refined macrophage‐responsive receptor set when receptor dynamics were analyzed along VSMC pseudotime. Candidate ligand‐receptor expression, SPP1–CD44 candidate axis, and NicheNet‐prioritized target program signatures were used to summarize candidate expression patterns and ligand‐associated target gene programs. The gene sets used for module scoring and bulk signature construction are provided in Supplementary Table S1.

Module scores were interpreted as summaries of transcriptional programs or candidate expression patterns. They were not interpreted as direct evidence of physical intercellular communication, functional ligand activity, or causal signaling.

Differential expression analysis was performed using the FindMarkers function in Seurat or Wilcoxon rank‐sum tests as appropriate. For macrophage analysis, highly inflammatory macrophages were compared with other macrophage subpopulations to identify genes associated with this inflammatory and stress‐response state. Pairwise comparisons of module scores among macrophage subpopulations were performed using Wilcoxon rank‐sum tests, and *p* values were adjusted for multiple comparisons using the Benjamini–Hochberg method. Effect sizes for Wilcoxon rank‐sum comparisons were calculated as rank‐biserial correlation to distinguish statistical significance from the magnitude of transcriptional differences. For VSMC analysis, marker genes and module scores were used to characterize phenotypic differences among VSMC subpopulations.

### 2.6 Functional enrichment analysis

Genes upregulated in highly inflammatory macrophages were subjected to Gene Ontology biological process and Kyoto Encyclopedia of Genes and Genomes (KEGG) enrichment analyses using clusterProfiler.^16^ Enrichment analysis was used to characterize the dominant biological programs associated with this macrophage state, with particular attention to inflammatory response, NF‐κB signaling, TNF response, leukocyte activation, cell adhesion, cytokine signaling, lipid‐associated pathways, and atherosclerosis‐related processes.

Because these enrichment results were derived from differentially expressed genes within the same single‐cell dataset, they were interpreted as functional annotations of the transcriptional state rather than independent mechanistic validation. Enriched terms and pathways were ranked according to statistical significance after multiple testing correction.

### 2.7 CellChat‐based candidate ligand‐receptor analysis

CellChat analysis was performed to prioritize candidate ligand‐receptor expression patterns among refined plaque cell states.^8^ The refined cell‐type annotation was used as the grouping variable. The human CellChat database was applied to identify ligand‐receptor pairs represented in the single‐cell transcriptomic data. CellChat‐reported aggregate probabilities were interpreted as model‐derived prioritization scores based on ligand and receptor expression and prior knowledge, rather than quantitative measurements of physical signaling strength or direct evidence of directional causality.

For the macrophage‐VSMC analysis, highly inflammatory macrophages and osteogenic/modulated VSMCs were selected as cell states of interest. Candidate ligand‐receptor pairs involving these cell states were extracted and visualized. Particular attention was paid to expression patterns related to inflammatory activation, extracellular matrix remodeling, growth factor signaling, and VSMC phenotypic modulation. These analyses were used to prioritize candidate ligand‐receptor expression patterns associated with inflammatory and remodeling‐related cell states, rather than to establish directional signaling or causal regulation between macrophages and VSMCs.

### 2.8 NicheNet ligand‐target prioritization analysis

NicheNet analysis was performed to prioritize candidate ligands associated with VSMC target gene programs.^9^ Highly inflammatory macrophages and osteogenic/modulated VSMCs were selected as the cell states of interest. Candidate ligands expressed in highly inflammatory macrophages and receptors expressed in osteogenic/modulated VSMCs were incorporated into the NicheNet ligand‐receptor and ligand‐target prior networks.

Ligand activity scores were calculated to prioritize candidate ligands associated with VSMC target gene programs. The resulting ligand‐target relationships were interpreted as computationally prioritized associations based on expression patterns and prior network information, not as direct evidence that macrophage ligands functionally regulate VSMC transcription in vivo.

Prioritized VSMC target genes were further used to construct a NicheNet‐prioritized target program score. This score was calculated across VSMC subpopulations to assess whether the ligand‐associated target program was enriched in osteogenic/modulated VSMCs. The same target gene set was subsequently applied to the external bulk transcriptomic dataset to examine its tissue‐level association with advanced plaques. These analyses were considered hypothesis‐generating and were not interpreted as independent validation of causal regulation between macrophage ligand expression and VSMC remodeling, particularly because ligand, receptor, and target gene programs may partially overlap with broader inflammatory, adhesion, extracellular matrix, and remodeling‐related transcriptional features.

### 2.9 VSMC pseudotime analysis

VSMC pseudotime analysis was performed using Slingshot.^10^ The VSMC subset was used as input after exclusion of the endothelial‐like contaminating population. Contractile VSMCs were selected as the primary starting state because they showed the highest expression of canonical contractile markers, including ACTA2, MYH11, TAGLN, CNN1, MYL9, and MYLK, whereas osteogenic/modulated VSMCs showed the strongest expression of remodeling‐associated and osteogenic/modulated markers, including FN1, VCAN, PDPN, COL11A1, integrin binding sialoprotein (IBSP), RUNX2, ALPL, and SPARC. The contractile‐to‐osteogenic/modulated direction was therefore used as a biologically guided pseudotime ordering model.

To reduce overinterpretation of pseudotime directionality, pseudotime was interpreted as transcriptional continuity among VSMC‐related states rather than direct lineage progression or temporal causality. For pseudotime‐related visualization and module‐score analysis, VSMC‐related cells were further summarized into three broad transcriptional states: contractile, VSMC‐like, and modulated states. A sensitivity analysis was performed using Slingshot without enforcing osteogenic/modulated VSMCs as the terminal state. The resulting pseudotime ordering was compared with the primary biologically guided ordering based on the distribution of VSMC‐related states, canonical marker gene gradients, and functional module scores. This analysis was used to assess whether the broad contractile‐to‐modulated transcriptional continuum was stable under a less constrained pseudotime setting.

To characterize transcriptional changes along pseudotime, VSMC functional module scores and representative marker genes were correlated with pseudotime using Spearman correlation analysis. The osteogenic/modulated score, refined macrophage‐responsive receptor score, contractile score, and selected genes related to VSMC remodeling and inflammatory responsiveness were examined along pseudotime. The refined macrophage‐responsive receptor score excluded intrinsic VSMC phenotypic switching markers when they were also used to define the osteogenic/modulated state, thereby reducing potential circularity in receptor dynamics analysis.

### 2.10 External bulk transcriptomic analysis

The GSE28829 bulk transcriptomic dataset was used to examine whether single‐cell‐derived inflammatory macrophage and VSMC remodeling programs were represented in human plaque progression at the tissue‐transcriptome level. Samples were classified into early and advanced plaque groups according to the original dataset annotation.

To reduce circularity and avoid confounding by general macrophage abundance or foam‐cell enrichment, a refined inflammatory macrophage signature was constructed using genes that were upregulated in highly inflammatory macrophages and consistent with the inflammatory and stress‐response transcriptional features of this state. Downregulated pan‐macrophage or foam‐cell‐associated genes, including APOE and TREM2, were not included in this refined signature. This approach was used to ensure that the bulk score reflected directionally consistent inflammatory and stress‐response features rather than a general macrophage or foam‐cell signal.

Bulk signature scores were calculated for the refined inflammatory macrophage signature, NF‐κB/TNF/IL1 signaling signature, inflammasome/NOD‐like receptor signaling signature, lysosome/autophagy signature, lipid‐atherosclerosis signature, osteogenic/modulated VSMC signature, candidate ligand‐receptor expression signature, SPP1‐CD44 candidate axis signature, and NicheNet‐prioritized target program signature. For signatures involving candidate ligand‐receptor or ligand‐target expression programs, the scores were interpreted as tissue‐level concordance of inflammatory, adhesion, extracellular matrix, and remodeling‐related transcriptional programs, not as independent validation of direct intercellular signaling.

For bulk transcriptomic analysis, gene expression values were standardized by gene using *Z*‐score transformation, and the average expression of genes within each signature was calculated as the corresponding signature score. When a gene was absent from the bulk expression matrix, it was excluded from score calculation. Signature scores were compared between early and advanced plaques using Wilcoxon rank‐sum tests. Correlations among signatures were assessed using Spearman correlation analysis. *p* values were adjusted for multiple comparisons using the Benjamini–Hochberg method where appropriate.

### 2.11 Statistical analysis and visualization

All statistical analyses were performed in R. Continuous variables were summarized using medians and interquartile ranges unless otherwise stated. Comparisons between two groups were performed using Wilcoxon rank‐sum tests. For comparisons involving multiple cell subpopulations or multiple signatures, *p* values were adjusted using the Benjamini–Hochberg method where appropriate.

For single‐cell module score comparisons, pairwise Wilcoxon rank‐sum tests were performed among macrophage or VSMC subpopulations, and adjusted *p* values were reported when multiple comparisons were conducted. Effect sizes for Wilcoxon rank‐sum comparisons were calculated as rank‐biserial correlation to distinguish statistical significance from the magnitude of transcriptional differences. Module‐score differences were interpreted according to both statistical significance and effect size rather than *p* values alone.

Correlations between module scores, signature scores, gene expression levels, and pseudotime were assessed using Spearman correlation analysis. Correlation coefficients were interpreted according to their magnitude, with weak correlations not considered evidence of strong biological coupling even when statistically significant in large single‐cell datasets. Therefore, correlation analyses were used to describe transcriptional associations rather than functional dependency or causal relationships.

A two‐sided *p* value < 0.05 was considered statistically significant unless otherwise specified. Data visualization was performed using Seurat, ggplot2, CellChat, NicheNet, Slingshot, and related R packages.

## 3 RESULTS

### 3.1 Single‐cell atlas of human atherosclerotic plaques

**FIGURE 1:**
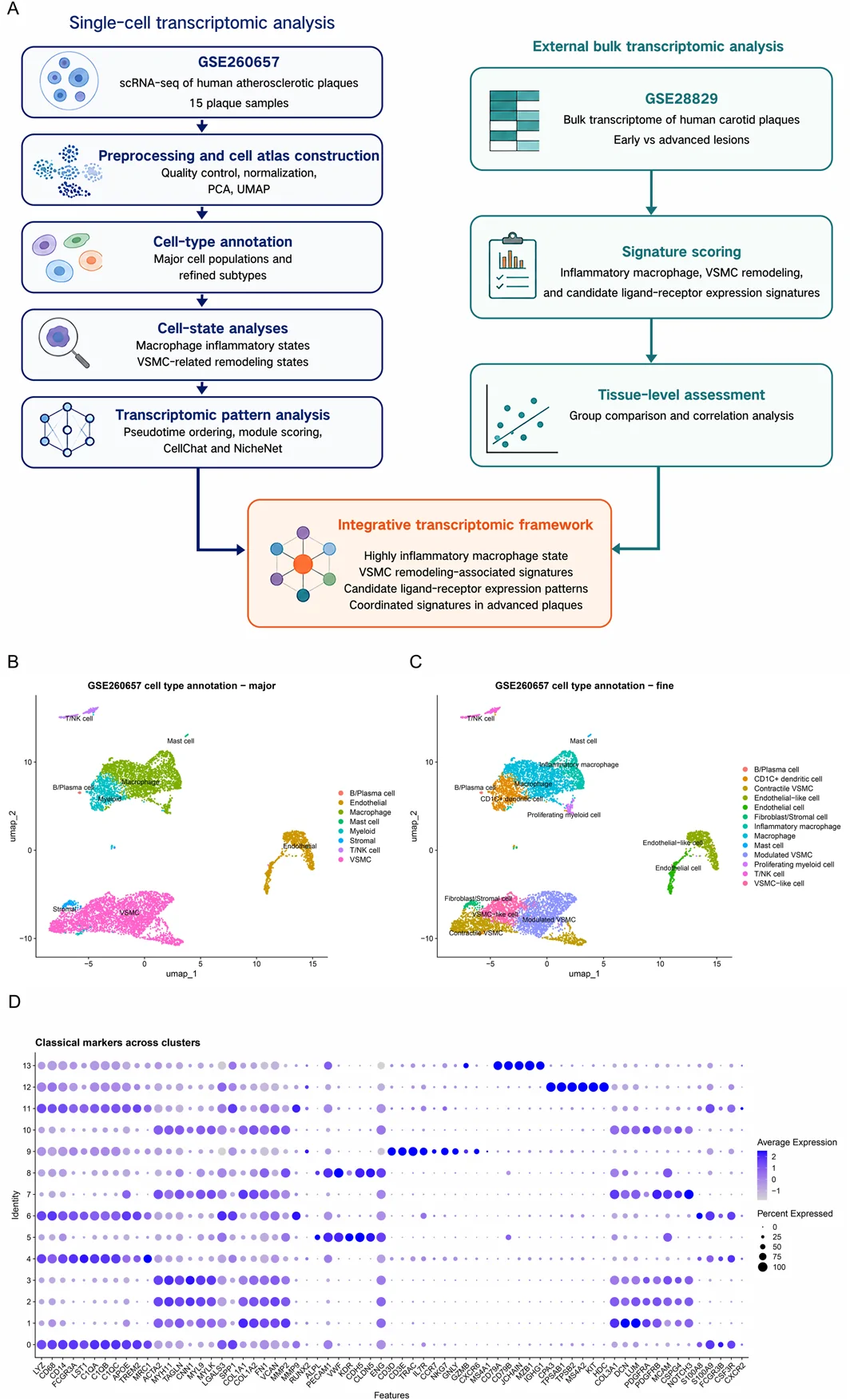
Study design and single‐cell transcriptomic atlas of human atherosclerotic plaques. (A) Overview of the integrative single‐cell RNA sequencing and bulk transcriptomic analysis. (B) Uniform manifold approximation and projection (UMAP) visualization of major cell types in GSE260657. (C) UMAP visualization of refined cell‐type annotations in GSE260657. (D) Dot plot showing canonical marker genes across annotated cell clusters.

After quality control, 7628 cells from human atherosclerotic plaque samples in GSE260657 were retained for downstream analysis. The overall study workflow is shown in Figure 1A. UMAP visualization and marker‐based annotation identified multiple major plaque cell populations, including VSMCs, macrophages, endothelial cells, myeloid cells, T/NK cells, stromal cells, mast cells, and B/plasma cells (Figure 1B,C). VSMCs and macrophages represented the two largest compartments, comprising 3392 and 2256 cells, respectively, followed by endothelial cells with 902 cells.

Canonical marker gene expression supported the cell‐type annotation. Macrophage‐related clusters expressed CD68, LYZ, LST1, C1QA, C1QB, and APOE; VSMC clusters expressed ACTA2, MYH11, TAGLN, and CNN1; endothelial cells expressed VWF, PECAM1, and CDH5; T/NK cells expressed CD3D, TRAC, NKG7, and GNLY; plasma cells expressed JCHAIN and MZB1; and mast cells expressed CPA3 and KIT (Figure 1D). This atlas provided the basis for subsequent macrophage‐ and VSMC‐focused analyses.

### 3.2 Identification of highly inflammatory macrophages by macrophage reclustering

**FIGURE 2:**
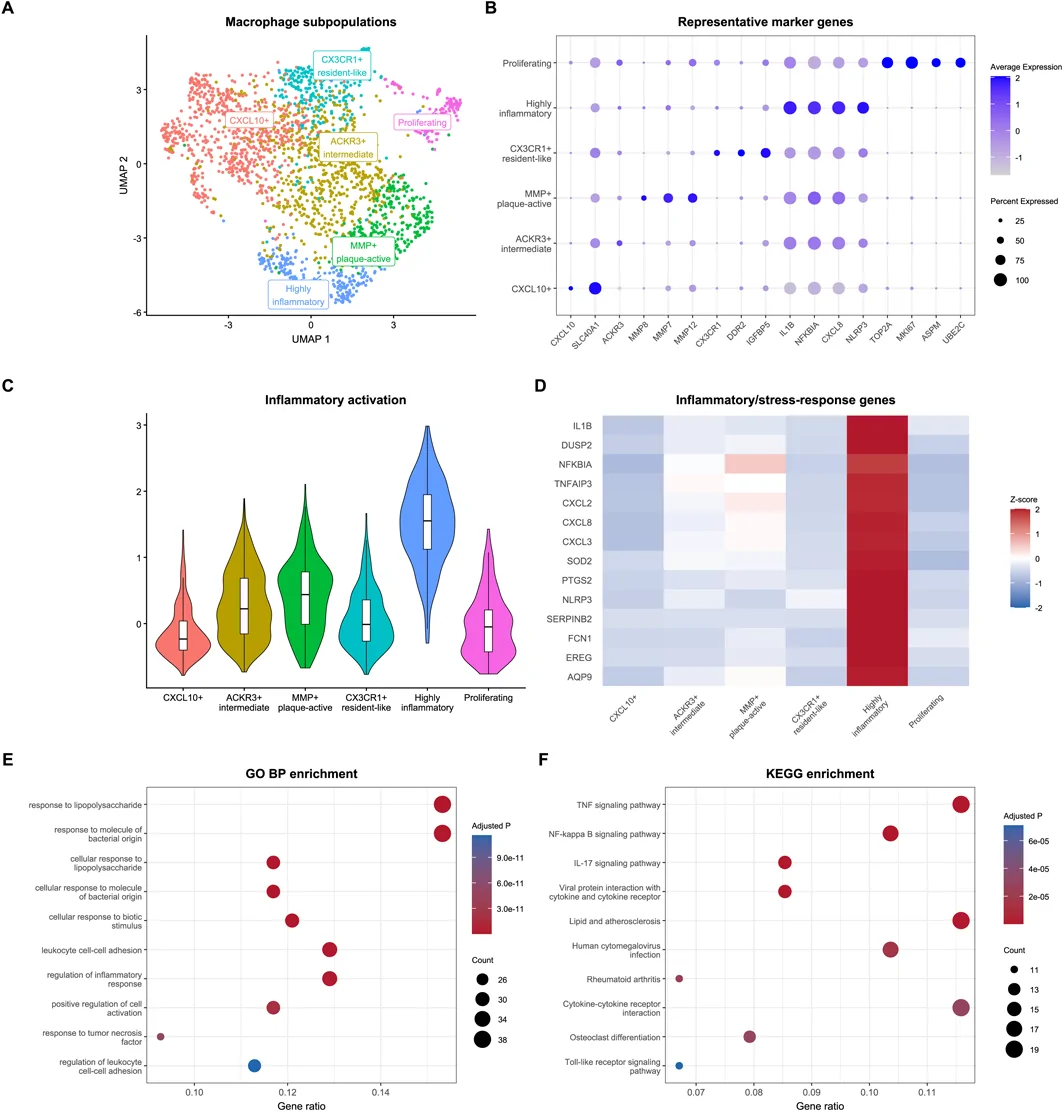
Identification and functional annotation of highly inflammatory macrophages. (A) Uniform manifold approximation and projection visualization of reclustered macrophage subpopulations. (B) Dot plot showing representative marker genes across macrophage subpopulations. (C) Inflammatory activation scores across macrophage subpopulations. (D) Heatmap showing selected inflammatory and stress‐response genes across macrophage subpopulations. (E) Gene Ontology biological process enrichment analysis of genes upregulated in highly inflammatory macrophages. (F) Kyoto Encyclopedia of Genes and Genomes enrichment analysis of genes upregulated in highly inflammatory macrophages.

To further characterize macrophage heterogeneity, 2256 macrophages were extracted and reclustered. Six macrophage subpopulations were identified, including CXCL10+ macrophages, ACKR3+ intermediate macrophages, MMP+ plaque‐active macrophages, CX3CR1+ resident‐like macrophages, highly inflammatory macrophages, and proliferating macrophages (Figure 2A). CXCL10+ macrophages represented the largest subgroup with 681 cells, followed by ACKR3+ intermediate macrophages with 657 cells, MMP+ plaque‐active macrophages with 322 cells, CX3CR1+ resident‐like macrophages with 260 cells, highly inflammatory macrophages with 214 cells, and proliferating macrophages with 122 cells.

Marker gene analysis showed distinct transcriptional features among macrophage subpopulations. CXCL10+ macrophages expressed CXCL10 and SLC40A1; MMP+ plaque‐active macrophages were characterized by MMP8, MMP7, and MMP12; CX3CR1+ resident‐like macrophages expressed CX3CR1, DDR2, and IGFBP5; and proliferating macrophages expressed TOP2A, MKI67, ASPM, and UBE2C (Figure 2B).

One macrophage cluster containing 214 cells showed the highest inflammatory activation score among macrophage subpopulations and was therefore annotated as highly inflammatory macrophages (Figure 2C). Differential expression analysis showed that this population upregulated inflammatory and stress‐response genes, including IL1B, DUSP2, NFKBIA, TNFAIP3, CXCL2, CXCL8, CXCL3, SOD2, PTGS2, NLRP3, SERPINB2, FCN1, EREG, and AQP9 (Figure 2D). These genes reflected a macrophage state dominated by cytokine response, chemokine expression, NF‐κB‐related activation, oxidative stress response, and inflammasome‐associated inflammatory features.

Together, macrophage reclustering identified a highly inflammatory macrophage state within human atherosclerotic plaques. This population was defined by inflammatory activation and stress‐response transcriptional features rather than by a distinct structural or communication‐specific identity.

### 3.3 Functional annotation of genes upregulated in highly inflammatory macrophages

GO BP and KEGG enrichment analyses were performed to annotate genes upregulated in highly inflammatory macrophages. GO BP enrichment highlighted inflammatory and immune‐response programs, including regulation of leukocyte cell‐cell adhesion, response to TNF, positive regulation of cell activation, regulation of inflammatory response, leukocyte cell–cell adhesion, cellular response to biotic stimulus, cellular response to molecules of bacterial origin, and response to lipopolysaccharide (Figure 2E).

KEGG analysis showed enrichment of pathways related to TNF signaling, NF‐κB signaling, IL‐17 signaling, lipid and atherosclerosis, cytokine–cytokine receptor interaction, toll‐like receptor signaling, viral protein interaction with cytokine and cytokine receptor, human cytomegalovirus infection, rheumatoid arthritis, and osteoclast differentiation (Figure 2F). These enrichment results were interpreted as functional annotations of the inflammatory and stress‐response features of this macrophage state rather than independent mechanistic validation.

### 3.4 VSMC reclustering reveals heterogeneous phenotypic states

To investigate VSMC heterogeneity, VSMC‐related cells were extracted and analyzed using the refined VSMC subtype annotation. Six VSMC‐related subpopulations were retained for downstream analysis: contractile VSMCs, PTHLH+ synthetic VSMCs, KRT7+ VSMC‐like cells, IFN‐responsive VSMCs, pericyte‐like mural cells, and osteogenic/modulated VSMCs.

Marker gene expression supported the annotation of these VSMC states. Contractile VSMCs highly expressed ACTA2, MYH11, TAGLN, CNN1, MYL9, and MYLK. PTHLH+ synthetic VSMCs showed higher expression of parathyroid hormone like hormone (PTHLH), KLF4, COL1A1, COL1A2, LGALS3, and FN1. KRT7+ VSMC‐like cells expressed KRT7, KRT18, EPCAM, and KRT8, whereas IFN‐responsive VSMCs were characterized by RSAD2, IFIT2, IFIT3, OAS1, ISG15, and MX1. Pericyte‐like mural cells expressed HIGD1B, SLCO1C1, CETP, PDGFRB, and NOTCH3. Osteogenic/modulated VSMCs were enriched for FN1, VCAN, PDPN, COL11A1, IBSP, RUNX2, ALPL, SPARC, and SPP1.

**FIGURE 3:**
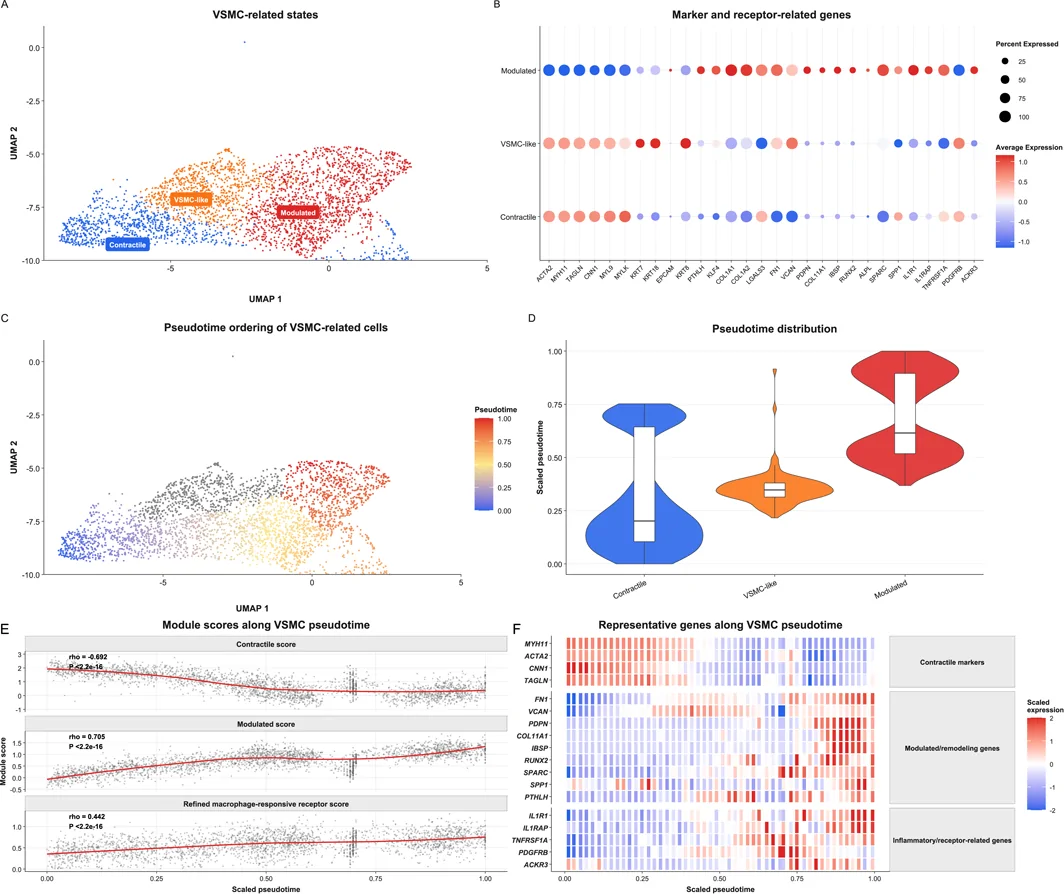
Vascular smooth muscle cell (VSMC)‐related state remodeling and pseudotime‐associated transcriptional patterns. (A) Uniform manifold approximation and projection visualization of broad VSMC‐related states. (B) Dot plot showing marker and receptor‐related genes across VSMC‐related states. (C) Pseudotime ordering of VSMC‐related cells. (D) Distribution of VSMC‐related states along scaled pseudotime. (E) Changes in contractile, modulated, and refined macrophage‐responsive receptor scores along scaled pseudotime. (F) Heatmap showing representative genes along VSMC pseudotime.

For pseudotime‐related visualization and module‐score analysis, VSMC‐related cells were further summarized into three broad transcriptional states: contractile, VSMC‐like, and modulated states (Figure 3A). Marker and receptor‐related gene expression across these broad states supported a contractile‐to‐modulated remodeling pattern, with contractile markers enriched in the contractile state and remodeling‐, osteogenic‐, and receptor‐related genes enriched toward the modulated state (Figure 3B). These findings indicated that plaque VSMC‐related cells contained multiple transcriptional states, including contractile, synthetic/intermediate, inflammatory‐responsive, mural‐like, and osteogenic/modulated phenotypes.

### 3.5 VSMC pseudotime analysis reveals a contractile‐to‐osteogenic/modulated transcriptional continuum

Slingshot pseudotime analysis was performed using contractile VSMCs as the biologically guided starting state and osteogenic/modulated VSMCs as the terminal reference state. The inferred ordering showed a transcriptional continuum from contractile VSMCs toward osteogenic/modulated VSMCs (Figure 3C). Contractile VSMCs were mainly distributed at the early stage of pseudotime, whereas modulated VSMC‐related states were enriched toward later pseudotime regions (Figure 3D). A sensitivity analysis performed without enforcing osteogenic/modulated VSMCs as the terminal state showed a broadly consistent ordering of VSMC‐related states. This supported the robustness of the major contractile‐to‐modulated transcriptional gradient, whereas the pseudotime direction was still interpreted cautiously as transcriptional continuity rather than direct lineage progression.

Functional module scores along pseudotime further supported this contractile‐to‐modulated transcriptional gradient. The contractile score showed a negative correlation with pseudotime (Spearman rho = −0.692 and *p* < 2.2 × 10^−16^), whereas the modulated score showed a positive correlation with pseudotime (rho = 0.705 and *p* < 2.2 × 10^−16^). The refined macrophage‐responsive receptor score also increased along pseudotime (rho = 0.442 and *p* < 2.2 × 10^−16^), indicating that VSMC‐related cells located toward the modulated end of the ordering displayed higher expression of selected inflammatory or remodeling‐associated receptor genes (Figure 3E).

At the single‐gene level, contractile markers, including MYH11, ACTA2, CNN1, and TAGLN, decreased along pseudotime. In contrast, remodeling and osteogenic/modulated genes, including FN1, VCAN, PDPN, COL11A1, IBSP, RUNX2, SPARC, SPP1, and PTHLH, increased along pseudotime. Several inflammatory or receptor‐related genes, including IL1R1, IL1RAP, TNFRSF1A, PDGFRB, and ACKR3, also showed increased expression toward the modulated end of the ordering (Figure 3F). These results supported a contractile‐to‐osteogenic/modulated transcriptional continuum associated with increased remodeling features and enhanced expression of selected macrophage‐responsive receptor genes, without implying direct lineage conversion or causal regulation by macrophages.

### 3.6 CellChat analysis prioritizes candidate ligand‐receptor expression patterns between highly inflammatory macrophages and osteogenic/modulated VSMCs

**FIGURE 4:**
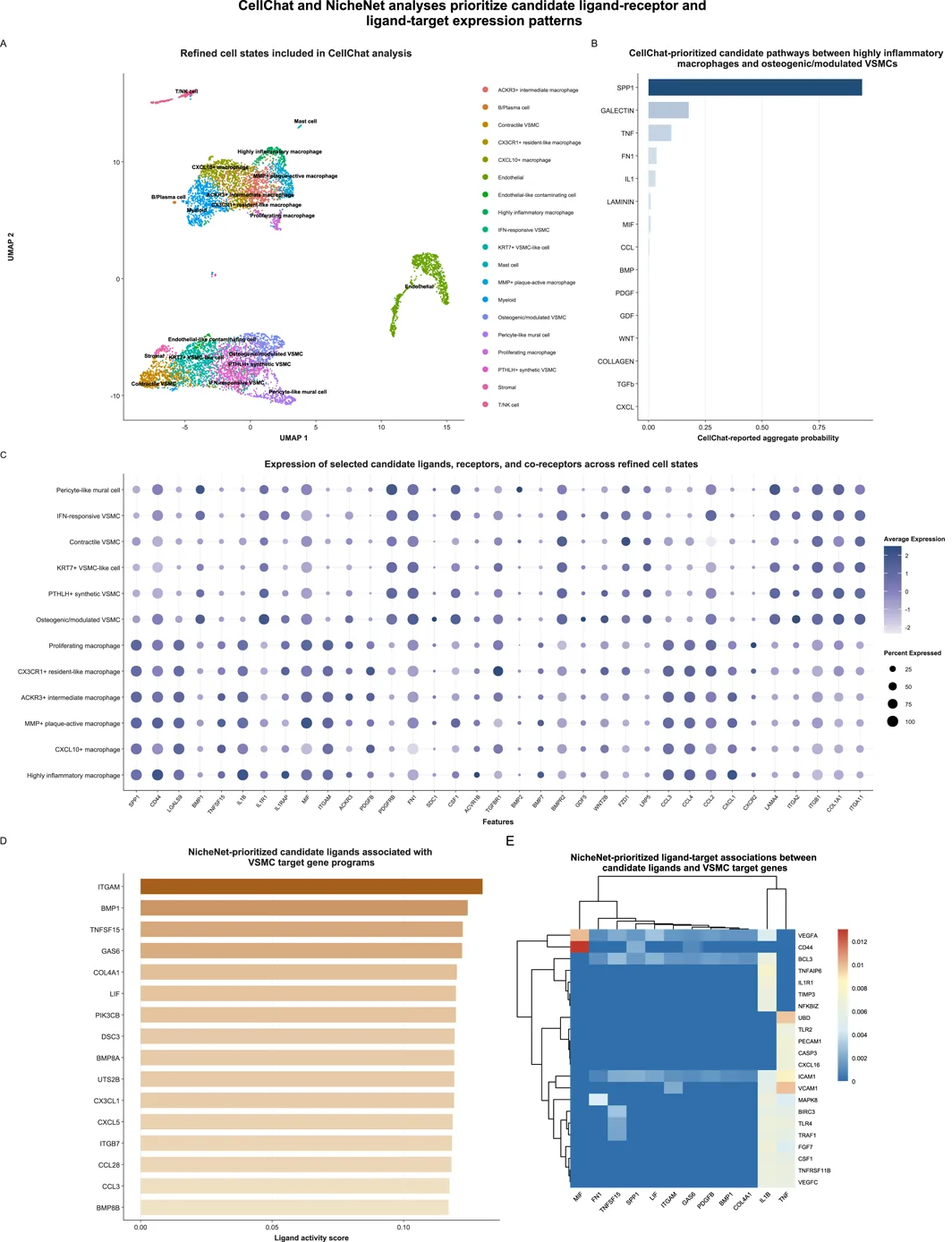
Candidate ligand‐receptor and ligand‐target expression patterns associated with highly inflammatory macrophages and osteogenic/modulated VSMCs. (A) Uniform manifold approximation and projection visualization of refined cell states included in CellChat analysis. (B) CellChat‐prioritized candidate pathways between highly inflammatory macrophages and osteogenic/modulated VSMCs. (C) Dot plot showing the expression of selected candidate ligands, receptors, and co‐receptors across refined cell states. (D) NicheNet‐prioritized candidate ligands associated with VSMC target gene programs. (E) Heatmap showing NicheNet‐prioritized ligand‐target associations between candidate ligands and VSMC target genes. VSMC, vascular smooth muscle cell.

To explore candidate ligand‐receptor expression patterns associated with inflammatory macrophage and VSMC remodeling states, CellChat analysis was performed using the refined cell‐type annotation (Figure 4A). When highly inflammatory macrophages and osteogenic/modulated VSMCs were evaluated as cell states of interest, several CellChat‐prioritized pathway‐level expression patterns were identified, including SPP1, GALECTIN, TNF, FN1, IL1, LAMININ, MIF, CCL, BMP, PDGF, GDF, WNT, COLLAGEN, TGFβ, and CXCL pathways (Figure 4B).

Among these CellChat‐prioritized pathways, SPP1 showed the highest CellChat‐reported aggregate probability with a probability of 0.940. Other relatively prominent pathways included GALECTIN with a probability of 0.176, TNF with 0.100, FN1 with 0.0352, IL1 with 0.0304, and MIF with 0.00984. Ligand‐receptor expression analysis showed that highly inflammatory macrophages and other macrophage subpopulations expressed candidate ligands such as SPP1, TNF, IL1B, MIF, PDGFB, FN1, CCL3, and CCL4. Osteogenic/modulated VSMCs and other VSMC‐related subpopulations expressed several candidate receptors or co‐receptors, including CD44, TNFRSF1A, IL1R1, IL1RAP, ACKR3, PDGFRB, SDC1, and ITGB1 (Figure 4C).

These results prioritized SPP1–CD44, TNF–TNFRSF1A, IL1B–IL1R1/IL1RAP, MIF–ACKR3, PDGFB–PDGFRB, and FN1–SDC1/ITGB1 as candidate ligand‐receptor expression patterns associated with highly inflammatory macrophages and osteogenic/modulated VSMCs. Because CellChat infers aggregate probabilities from ligand‐receptor expression and prior knowledge, these findings should be interpreted as computationally prioritized expression patterns rather than direct evidence of physical signaling strength, directional intercellular signaling, or causal VSMC remodeling.

### 3.7 NicheNet prioritizes ligand‐associated VSMC target gene programs

To further explore candidate links between macrophage‐expressed ligands and VSMC transcriptional programs, NicheNet analysis was performed. Ligand activity analysis and ligand‐target prioritization identified multiple candidate macrophage‐expressed ligands associated with VSMC target genes, including ITGAM, BMP1, TNFSF15, GAS6, COL4A1, LIF, PIK3CB, DSC3, BMP8A, UTS2B, IL1B, PDGFB, FN1, SPP1, TNF, and MIF (Figure 4D).

The NicheNet‐prioritized ligand‐target association matrix showed that several candidate ligands were linked to VSMC target genes involved in inflammatory activation, adhesion, receptor signaling, and remodeling‐associated responses. These target genes included CD44, IL1R1, TNFAIP6, ICAM1, VCAM1, TLR2, TLR4, BCL3, and NFKBIZ (Figure 4E).

To evaluate whether the prioritized target gene program was represented in VSMC remodeling states, a NicheNet‐prioritized target program score was calculated. At the single‐cell level, this score was highest in osteogenic/modulated VSMCs. The median score in osteogenic/modulated VSMCs was 0.140, compared with −0.129 in other VSMC subpopulations, with a median difference of 0.270 (*p* < 2.2 × 10^−16^). In addition, the target program score increased along VSMC pseudotime, suggesting that this ligand‐associated program was enriched toward the osteogenic/modulated end of the ordering. These results should be interpreted as computationally prioritized ligand‐target associations and not as direct evidence that macrophage‐derived ligands functionally regulate VSMC transcription in vivo.

### 3.8 External bulk analysis shows coordinated inflammatory and remodeling signatures in advanced plaques

To evaluate whether the single‐cell‐derived inflammatory and remodeling programs were reflected at the tissue level, external bulk transcriptomic analysis was performed using GSE28829, which contains early and advanced human atherosclerotic plaques. Signature scores were calculated for the refined inflammatory macrophage signature, NF‐κB/TNF/IL1 signaling, inflammasome/NOD‐like receptor signaling, lysosome/autophagy, lipid‐atherosclerosis, osteogenic/modulated VSMC signature, candidate ligand‐receptor expression signature, SPP1–CD44 candidate axis signature, and NicheNet‐prioritized target program signature.

**FIGURE 5:**
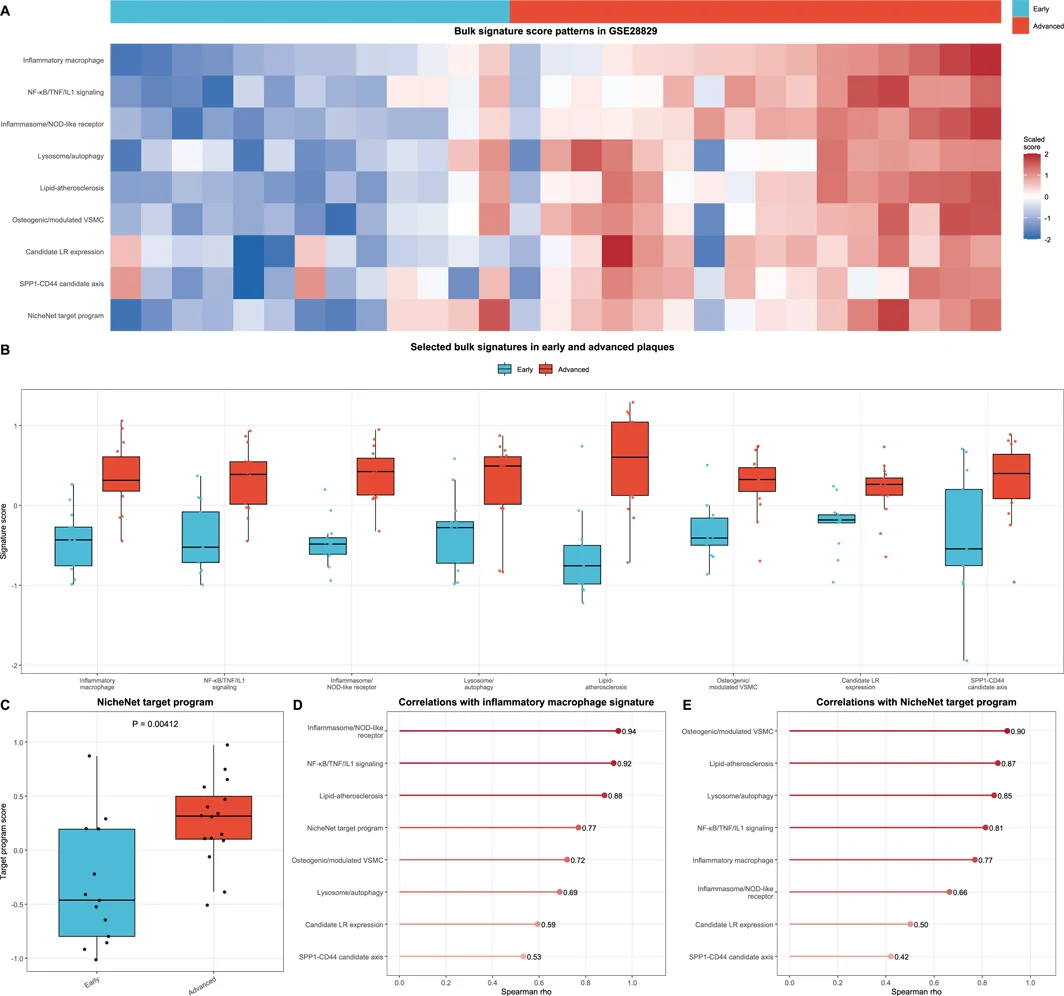
Coordinated inflammatory and remodeling signatures in advanced plaques from GSE28829. (A) Heatmap showing bulk signature score patterns in early and advanced plaques. (B) Comparison of selected inflammatory, remodeling, and candidate ligand‐receptor expression signatures between early and advanced plaques. (C) Comparison of the NicheNet target program score between early and advanced plaques. (D) Spearman correlations between the inflammatory macrophage signature and other bulk signatures. (E) Spearman correlations between the NicheNet target program and other bulk signatures.

Heatmap visualization showed that multiple inflammatory macrophage‐, lipid/lysosome‐, osteogenic VSMC‐, candidate ligand‐receptor‐, SPP1–CD44‐, and NicheNet target‐related signatures were more prominently represented in advanced plaques than in early plaques (Figure 5A). Group comparison further showed higher scores for selected inflammatory and remodeling‐associated signatures in advanced plaques, including the refined inflammatory macrophage signature, NF‐κB/TNF/IL1 signaling, inflammasome/NOD‐like receptor signaling, lysosome/autophagy, lipid‐atherosclerosis, osteogenic/modulated VSMC signature, candidate ligand‐receptor expression signature, and SPP1–CD44 candidate axis signature (Figure 5B).

The NicheNet‐prioritized target program score was also higher in advanced plaques than in early plaques (*p* = 0.00412), indicating that this ligand‐associated VSMC target program was represented in bulk plaque transcriptomes (Figure 5C). Correlation analysis showed positive associations among inflammatory macrophage‐, VSMC remodeling‐, lipid/lysosome‐, candidate ligand‐receptor‐, SPP1–CD44‐, and NicheNet target‐related signatures (Figure 5D,E).

Together, these bulk transcriptomic results indicated that inflammatory macrophage‐related signatures and VSMC remodeling‐related signatures were concurrently more prominent in advanced plaques. Because several signatures shared inflammatory, adhesion, extracellular matrix, and remodeling‐associated genes, their concordant enrichment and positive correlations should not be interpreted as independent evidence of directional macrophage–VSMC signaling or causal intercellular regulation. Rather, these findings indicate tissue‐level concordance between inflammatory activation and structural remodeling programs during plaque progression.

## 4 DISCUSSION

In this study, we integrated human plaque scRNA‐seq data with an external bulk transcriptomic dataset to characterize inflammatory macrophage activation and VSMC remodeling in human atherosclerosis. The single‐cell analysis identified a highly inflammatory macrophage state marked by IL1B, NFKBIA, TNFAIP3, CXCL2, CXCL8, NLRP3, PTGS2, SERPINB2, and related inflammatory or stress‐response genes. VSMC‐related cells showed a broad contractile‐to‐osteogenic/modulated transcriptional continuum accompanied by decreased contractile marker expression and increased remodeling‐, extracellular matrix‐, osteogenic‐, and receptor‐related programs. CellChat and NicheNet analyses further prioritized candidate ligand‐receptor and ligand‐associated target gene expression patterns linking highly inflammatory macrophages with osteogenic/modulated VSMCs. In the external bulk dataset GSE28829, inflammatory macrophage, VSMC remodeling, candidate ligand‐receptor expression, SPP1‐CD44 candidate axis, and NicheNet‐prioritized target program signatures were more prominent in advanced plaques. Together, these findings describe coordinated inflammatory and remodeling transcriptional programs in advanced human atherosclerotic plaques.

A major finding of this study was the identification of a highly inflammatory macrophage state within human plaques. This population was characterized by canonical inflammatory and stress‐response genes, including IL1B, NFKBIA, TNFAIP3, CXCL2, CXCL8, NLRP3, and PTGS2. Functional enrichment analysis further linked this state to TNF signaling, NF‐κB signaling, IL‐17 signaling, toll‐like receptor signaling, cytokine–cytokine receptor interaction, and lipid and atherosclerosis‐related pathways. These findings are consistent with the established role of macrophage inflammatory activation in plaque progression.^17^, ^18^ Importantly, this macrophage state should not be overinterpreted as a newly defined macrophage lineage or a structurally distinct macrophage phenotype. The value of this analysis lies in placing this inflammatory macrophage state within an integrated framework that also includes VSMC remodeling, candidate ligand‐receptor expression patterns, and bulk plaque progression‐associated signatures.

VSMC phenotypic remodeling is a central feature of atherosclerotic plaque development.^19^ In this study, VSMC‐related cells included contractile, synthetic/intermediate, IFN‐responsive, mural‐like, and osteogenic/modulated states. Pseudotime ordering showed a broad contractile‐to‐modulated transcriptional continuum, with decreasing expression of contractile markers and increasing expression of remodeling and osteogenic/modulated genes, including FN1, VCAN, PDPN, COL11A1, IBSP, RUNX2, SPARC, and SPP1. These gene‐expression patterns are consistent with previously reported VSMC phenotypic modulation, extracellular matrix remodeling, and osteogenic/calcification‐related programs in atherosclerotic lesions.^7^, ^20^ The refined macrophage‐responsive receptor score also increased toward the modulated end of the ordering, suggesting that VSMC‐related cells with stronger remodeling features also expressed selected receptors potentially responsive to inflammatory or remodeling‐associated cues. This pseudotime analysis should be interpreted as transcriptional ordering among VSMC‐related states, without implying lineage conversion, temporal sequence, or macrophage‐driven VSMC remodeling.

The CellChat and NicheNet analyses prioritized candidate expression patterns that may help connect inflammatory macrophage states with VSMC remodeling states at the transcriptomic level. CellChat highlighted pathway‐level patterns involving SPP1, TNF, IL1, MIF, PDGF, FN1, and other ligand‐receptor systems. SPP1 encodes osteopontin, a secreted phosphoprotein detected in macrophages, VSMCs, and endothelial cells in human atherosclerotic plaques.^21^ Osteopontin has also been implicated in vascular inflammation, cell adhesion, immune activation, and calcification‐related remodeling.^22^, ^23^ In the present analysis, SPP1–CD44, TNF–TNFRSF1A, IL1B–IL1R1/IL1RAP, MIF–ACKR3, PDGFB–PDGFRB, and FN1–SDC1/ITGB1 emerged as candidate ligand‐receptor expression patterns associated with highly inflammatory macrophages and osteogenic/modulated VSMCs. MIF‐related signaling has been associated with inflammatory cell recruitment and vascular inflammation.^24^ PDGF signaling is closely related to vascular wall cell activation and smooth muscle cell accumulation.^25^ FN1‐related adhesion signaling may participate in extracellular matrix remodeling during VSMC phenotypic modulation.^26^, ^27^

NicheNet further prioritized candidate ligands and VSMC target genes involved in inflammatory activation, adhesion, receptor signaling, and remodeling‐associated responses. These target genes included CD44, IL1R1, TNFAIP6, ICAM1, VCAM1, TLR2, TLR4, BCL3, and NFKBIZ. These ligand‐receptor and ligand‐target patterns are biologically plausible in the context of plaque inflammation and remodeling. However, CellChat and NicheNet are based on transcriptomic expression and prior interaction networks.^8^, ^9^ Their outputs do not demonstrate physical cell–cell signaling, ligand secretion, receptor activation, downstream signaling, or causal regulation in vivo.^28^ Therefore, the candidate ligand‐receptor and ligand‐target patterns identified here should be considered hypothesis‐generating priorities for future spatial, experimental, and functional validation.

The external bulk transcriptomic analysis provided tissue‐level context for the single‐cell‐derived signatures. In GSE28829, advanced plaques showed higher inflammatory macrophage, NF‐κB/TNF/IL1 signaling, inflammasome/NOD‐like receptor, lysosome/autophagy, lipid‐atherosclerosis, osteogenic/modulated VSMC, candidate ligand‐receptor expression, SPP1–CD44 candidate axis, and NicheNet‐prioritized target program signatures. These findings indicate that inflammatory and remodeling‐related transcriptional programs identified in the single‐cell analysis are also represented at the bulk plaque level during disease progression.^29^, ^30^ Correlations among these signatures further suggest coordinated inflammatory and structural remodeling features in advanced plaques. However, several signatures include overlapping inflammatory, adhesion, extracellular matrix, and remodeling‐associated genes. Therefore, their concordant enrichment and positive correlations should not be interpreted as independent evidence of directional macrophage–VSMC signaling or causal intercellular regulation. These results instead reflect tissue‐level concordance of related inflammatory and remodeling transcriptional programs.

Several limitations should be acknowledged. First, this study was based on publicly available transcriptomic datasets, and the sample size, plaque‐region information, and clinical metadata were limited by the original studies. Second, scRNA‐seq cannot directly resolve spatial organization, physical cell–cell contact, ligand secretion, receptor activation, or downstream signaling activity. Third, CellChat and NicheNet infer candidate associations from gene expression and prior knowledge networks; therefore, their outputs require spatial, biochemical, and functional validation before being interpreted as true intercellular signaling events. Fourth, pseudotime analysis describes transcriptional ordering among cell states rather than true temporal progression or lineage conversion.^10^ Finally, bulk transcriptomic analysis reflects mixed tissue‐level signals and cannot distinguish whether correlated signatures arise from direct cellular interactions, shared inflammatory microenvironments, altered cell composition, or coordinated plaque remodeling. Future studies integrating spatial transcriptomics, multiplex imaging, perturbation experiments, and cell‐type‐specific validation will be needed to test the candidate ligand‐receptor and ligand‐target programs prioritized in this analysis.

## 5 CONCLUSION

In this study, we integrated human atherosclerotic plaque scRNA‐seq data with an external bulk transcriptomic dataset to characterize inflammatory macrophage activation and VSMC remodeling‐related transcriptional states. We identified a highly inflammatory macrophage state characterized by inflammatory activation, cytokine‐response, stress‐response, lysosome/autophagy‐related, and lipid‐atherosclerosis‐associated transcriptional features. VSMC pseudotime analysis revealed a broad transcriptional continuum from contractile VSMCs toward osteogenic/modulated VSMCs accompanied by increased expression of remodeling‐associated genes and selected inflammatory or remodeling‐associated receptor genes. CellChat and NicheNet analyses further prioritized candidate ligand‐receptor and ligand‐associated VSMC target gene expression patterns involving SPP1–CD44, TNF–TNFRSF1A, IL1B–IL1R1/IL1RAP, MIF–ACKR3, PDGFB–PDGFRB, and FN1–SDC1/ITGB1. External bulk transcriptomic analysis in GSE28829 showed that inflammatory macrophage‐, osteogenic/modulated VSMC‐, candidate ligand‐receptor expression‐, SPP1‐CD44 candidate axis‐, and NicheNet‐prioritized target program‐related signatures were more prominent in advanced plaques and positively correlated with each other. Overall, these findings support a coordinated transcriptomic association between inflammatory macrophage activation and VSMC remodeling in human atherosclerosis and provide hypothesis‐generating candidate ligand‐receptor and ligand‐target programs for future spatial and functional validation.

## AUTHOR CONTRIBUTIONS

**Xinyu Dong**: Conceptualization; methodology; formal analysis; data curation; visualization; funding acquisition; writing—original draft. **Xuesong Hu**: Data curation; formal analysis; visualization; writing—review and editing. **Qi Liu**: Data curation; formal analysis; validation; writing—review and editing. **Yuhao Dong**: Validation; formal analysis; visualization; writing—review and editing. **Yan Xuan**: Investigation; data interpretation; gene set curation; writing—review and editing. **Muning Wang**: Investigation; data interpretation; gene set curation; writing—review and editing. **Siyao Chang**: Literature review; resources; writing—review and editing. **Tianyi Lu**: Literature review; figure organization; writing—review and editing. **Zhiyang Han**: Conceptualization; supervision; project administration; funding acquisition; writing—review and editing. All authors read and approved the final manuscript.

## CONFLICT OF INTEREST STATEMENT

The authors declare no conflicts of interest.

## ETHICS STATEMENT

This study was based entirely on publicly available datasets. No new human or animal experiments were performed; therefore, additional ethical approval was not required.

## CONSENT FOR PUBLICATION

Not applicable.

## Supporting information

Table S1

## Data Availability

The datasets analyzed in this study are available in the GEO database under accession numbers GSE260657 and GSE28829.
